# 737 Prevalence and Outcomes of HIV Infection Among Burn Patients at a Single Institution

**DOI:** 10.1093/jbcr/irac012.290

**Published:** 2022-03-23

**Authors:** Sanja Sljivic, Lori Chrisco, Rabia Nizamani, Booker King, Felicia Williams

**Affiliations:** University of North Carolina - Chapel Hill, Chapel Hill, North Carolina; North Carolina Jaycee Burn Center, Chapel Hill, North Carolina; UNC Jaycee Burn Center, Chapel Hill, North Carolina; UNC Jaycee Burn Center, Chapel Hill, North Carolina; UNC Jaycee Burn Center, Chapel Hill, North Carolina

## Abstract

**Introduction:**

Burn injuries are a significant cause of morbidity and mortality worldwide. Pre-existing conditions may further complicate a patient’s outcome and delay wound healing. Human immunodeficiency virus (HIV) remains an ongoing problem globally and contributes to the morbidity of patients with burn injuries. According to the Centers for Disease Control and Prevention (CDC), the prevalence of individuals with HIV in the United States was 1.2 million in 2018. Burn patients and those with desquamating skin disorders are already in an immunocompromised state, and thus, the effect of HIV on the healing and recovery process can be significant. The objective of this study was to evaluate the prevalence and outcomes of HIV-positive patients admitted to our Burn Center.

**Methods:**

This was a single-site, retrospective review using our institutional Burn Center registry. All adult patients (18 years or older) admitted to our Burn Center between July 1, 2010 and June 30, 2020 who were HIV-positive were included in this study. All adult patients who were HIV-negative and admitted during the same period were included for comparative purposes. Variables of interest included demographics, burn mechanism, length of stay (LOS), cost of hospitalization, and mortality.

**Results:**

There were 32 HIV-positive burn patients and 16 HIV-positive patients with desquamating skin disorders (e.g., Stevens-Johnson syndrome/Toxic Epidermal Necrolysis). For the burn group, the mean age was 46.9 years +/- 10.6 years, and the mean total body surface area (TBSA) involvement was 3.2% +/- 4.2%. The mean LOS among HIV-positive burn patients was 9.13 days +/- 17.73 days, and the mean cost of hospitalization was $54,613. For the desquamating skin disorders group, the mean age was 47.1 years +/- 13.9 years, and the mean TBSA was 16.2% +/- 29.0%. The mean LOS was 17.25 days +/- 25.26 days, and the mean cost of hospitalization was $138,358. In terms of overall hospital mortality, there were no deaths among HIV-positive burn patients; however, the mortality was 25% among HIV-positive patients with desquamating skin disorders (n = 4). When both groups were compared to HIV-negative patients, overall hospital mortality remained higher among HIV-positive patients with desquamating skin disorders.

**Conclusions:**

Management of HIV-positive burn patients presents a unique challenge for clinicians due to the immunocompromised state of this patient population. The challenge may even be more pronounced in HIV-positive patients with desquamating skin disorders as demonstrated in this study.

